# CHOT STUDENT REPRESENTATIVE

**DOI:** 10.1093/geroni/igz038.1548

**Published:** 2019-11-08

**Authors:** Neeraj Puro

**Affiliations:** University of Alabama at Birmingham, UAB, Alabama, United States

## Abstract

As the Senior Student Representative for the Social Research, Policy, and Practice Section for GSA one of the goals is to organize a symposium that would be beneficial for students and junior researchers. In this section, we will have a student who was involved in CHOT to speak about his experience. Neeraj Puro is a doctoral candidate who has been involved in several CHOT projects during his academic career. The CHOT has helped fund his doctoral work and allowed him to travel and present at more conferences. The projects that he was involved in helped him with early publications and assisted when he was out in the career market. He will speak about his experience; what he learned, and his opinion about the process. He will reflect on this program from a student/junior researcher’s perspective and how it has influenced his doctoral journey and the beginning of his career.

